# Phase I pharmacokinetic and pharmacodynamic study of the prenyl transferase inhibitor AZD3409 in patients with advanced cancer

**DOI:** 10.1038/sj.bjc.6604402

**Published:** 2008-05-27

**Authors:** N M G M Appels, M J Bolijn, K Chan, T C Stephens, G Hoctin-Boes, M Middleton, J H Beijnen, J S de Bono, A L Harris, J H M Schellens

**Affiliations:** 1 Department of Pharmacy and Pharmacology, Slotervaart Hospital/The Netherlands Cancer Institute, Louwesweg 6, Amsterdam 1066 EC, The Netherlands; 2 The Netherlands Cancer Institute/Antoni van Leeuwenhoek Hospital, Plesmanlaan 121, Amsterdam 1066 CX, The Netherlands; 3 AstraZeneca, Alderley Park, Macclesfield, Cheshire SK10 4TG, UK; 4 Cancer Research UK Medical Oncology Unit, Churchill Hospital, Headington, Oxford OX37L, UK; 5 Beta Faculty, Department of Pharmaceutical Sciences, Division of Drug Toxicology, Department of Biomedical Analysis, Utrecht University, PO Box 80082, Utrecht 3508 TB, The Netherlands; 6 Royal Marsden Hospital, Downs Road, Sutton, Surrey SM25PT, UK

**Keywords:** AZD3409, prenyl transferase inhibitor, biological effect study

## Abstract

AZD3409 is an orally active double prodrug that was developed as a novel dual prenyltransferase inhibitor. The formation of the active metabolite AZD3409 acid is mediated by esterases in plasma and cells. The aim of this phase I study was to determine the maximum tolerated dose, toxicities, pharmacokinetics and pharmacodynamics of AZD3409. AZD3409 was administered orally to patients with advanced solid malignancies using an interpatient dose-escalation scheme starting at 500 mg AZD3409 once daily. Twenty-nine patients were treated at seven dose levels. The MTD of part A was defined as 750 mg b.i.d. in the fasted state. Adverse events were mainly gastrointestinal and the severity was on average mild to moderate and reversible. The dose-limiting toxicities were vomiting, diarrhoea and uncontrolled nausea. Pharmacokinetic studies of the prodrug and the active metabolite indicated dose proportionality. Pharmacodynamic studies showed that farnesyltransferase (FTase) was inhibited at all dose levels. In conclusion, chronic oral dosing with AZD3409 is feasible and results in significant inhibition of FTase activity. Pharmacodynamic studies revealed that the maximal FTase inhibition, estimated at 49±11%, appeared to be reached at AZD3409 acid plasma concentrations at which the occurrence of drug-related toxicity was low. This study supports the rationale to implement biological effect studies in clinical trials with biologically active anticancer drugs to define optimal dosing regimens.

AZD3409 is a novel dual prenyl transferase inhibitor (DPTI) developed for the treatment of cancer. Prenyl transferases comprise a group of enzymes that are responsible for the posttranslational addition of a farnesyl or geranylgeranyl moiety to proteins that share a common C-terminal sequence, the so-called CAAX-box (Casey and Seabra, 1996). One of these proteins is the oncoprotein Ras. Ras exists in three isoforms: H-Ras, N-Ras and K-Ras (with splice variants Ki4A and Ki4B). In human cancer, the Ki4B-Ras protein is the most commonly mutated Ras protein, and, therefore, it is an attractive target for intervention in tumour development (Bos, 1989). In more recent years, it has been shown that other farnesylated proteins such as RhoB, Rheb and centromere proteins also play a role in tumour development and are, therefore, interesting targets in cancer drug development (Tamanoi *et al*, 2001; Delarue *et al*, 2007).

Initial drug development focused on the inhibition of farnesylation (Crul *et al*, 2001; Brunner *et al*, 2003). However, blockage of farnesylation does not result in unprenylated Ki4B-Ras. Instead, geranylgeranyl transferase (GGTase-1) takes over the posttranslational modification of Ki4B-Ras, thereby preserving its function in cell growth processes (Whyte *et al*, 1997). This could be a possible underlying mechanism to the low-to-modest activity of farnesyltransferase (FTase) inhibitors in clinical phase II and III studies (Sebti and Adjei, 2004). Clinical trials exploring FTase inhibitors in combination with other anticancer drugs are still in progress (Leary *et al*, 2007). Second-generation prenyl transferase inhibitors have inhibitory properties on both FTase and GGTase-1 and are, therefore, called DPTIs. It is expected that these drugs can completely inhibit prenylation of Ki4B-Ras resulting in increased clinical antitumour effects compared with FTase inhibitors.

AZD3409, developed by AstraZeneca, is an orally administered DPTI that is administered as a double prodrug (Figure 1). In plasma, it is rapidly converted to the circulating thiol-ester prodrug. This is referred to as AZD3409 ester. The AZD3409 ester can penetrate into the cell where a thiol-acid moiety is formed under the action of esterases. This is the active drug and is referred to as AZD3409 acid. It is a potent inhibitor of FTase (*K*_i_<1 nM) and a moderately potent inhibitor of GGTase-1 (*K*_i_=8 nM) using Ki4B-Ras as a substrate (Stephens *et al*, 2003).

In preclinical studies in mice bearing xenografts of H-Ras-transformed mouse 3T3 fibroblasts (Hras5), and of 253J-BV or RT-112 urothelial cells, significant reduction of tumour weight was observed upon treatment with AZD3409 (Stephens *et al*, 2003; Kelly *et al*, 2005). In mice bearing Hras5 tumours, growth inhibition was correlated with inhibition of FTase activity. These results provided a preclinical proof of the concept. Additionally, biomarker assays were set up to determine the biological effect of AZD3409 (unpublished data). The HDJ-2 and Rap1a prenylation levels were chosen as suitable surrogate end points for farnesylation and geranylgeranylation, respectively. Preclinical proof of concept and the availability of surrogate parameter assays were considered to be a good starting point for a phase I clinical study. The first trial with AZD3409 was performed in healthy male volunteers. The main toxicities were diarrhoea (dose limiting at 2500 mg once daily), nausea, dizziness, abdominal pain and vomiting. The next lower dose of 1750 mg was defined as the maximum tolerated single dose in this group.

Here, we present our results of a phase I trial of AZD3409 in patients with advanced cancer using a continuous daily dosing scheme. The objectives of the study were (1) to investigate the safety and tolerability of multiple oral doses of AZD3409 in patients with advanced solid malignancies; (2) to investigate the pharmacokinetic (PK) profile of AZD3409 ester and AZD3409 acid; and (3) to investigate the degree of FTase inhibition after administration of AZD3409 in surrogate and tumour tissue.

## PATIENTS AND METHODS

### Patient population

This phase I dose-escalating study of AZD3409 was a multicentre study. Patients were eligible if they had histologically or cytologically confirmed diagnosis of a solid malignant tumour. Other eligibility criteria included a WHO performance status of 0–2 and age ⩾18 years. Women with childbearing potential were eligible when using contraceptive methods as defined by a method of a failure rate less than 1% per year when used consistently and correctly.

Previous chemotherapy and radiation therapy had to be discontinued for at least 4 weeks before entry into the study. All patients had to have acceptable bone marrow function, defined by neutrophil counts ⩾3.5 × 10^9^/l, platelets ⩾100 × 10^9^/l and Hb⩾5.6 mmol/l; and adequate hepatic and renal function defined as creatinine clearance ⩾50 ml min^−1^, total bilirubin ⩽1.5 times the normal upper limit, and ASAT and ALAT ⩽2.5 times the normal upper limit (or ⩽5 times the normal upper limit in case of hepatic metastases). The Medical Ethics Committee of all hospitals approved the study protocol and all patients had to give written informed consent prior to start.

### Treatment plan and study design

AZD3409 was supplied by AstraZeneca as 250 mg capsules. It was given once daily, after fasting from food and liquid for at least 1 h. During the study, the dosing regimen was switched from once daily to twice daily (12 h apart), because PK/pharmacodynamic (PD) modelling based on data from a single ascending dose volunteer study predicted that b.i.d. dosing would provide prolonged FTase inhibition over a 24-h time period.

The study was divided into two parts. Part A was an open-label dose-escalation study to define the maximum tolerated dose (MTD). Part B was an enrichment phase in female patients having advanced breast cancer for whom there was no standard therapy available.

AZD3409 was administered continuously starting on day 1 of each cycle of 21 days. A modified Fibonacci schedule was used for dose escalation, starting from 500 mg AZD3409. This dose was predicted to achieve a steady-state exposure of approximately 25% of the MTD observed in the healthy volunteer study. At least three patients were treated at each dose level. The occurrence of a DLT in a patient during the first cycle led to enrolment of three additional patients. Patients with progressive disease were removed from the study and patients who were removed within the first cycle of the study for reasons other than drug-related toxicity were replaced.

Once the MTD was defined in part A an open-label safety run-in phase commenced, with patients in part B being dosed in the fed state, initially at 250 mg b.i.d. At this point, a decision to stop the clinical evaluation of AZD3409 was made and both the dose escalation and the enrichment phase were not progressed.

### Patient evaluation

Pretreatment evaluation included a complete medical history and physical examination. Measurable and evaluable disease was documented before the start of the study and during the study as a basis for the assessment of clinical activity of the treatment. Tumour response was recorded according to RECIST criteria (Therasse *et al*, 2000). Toxicity was monitored at each visit. All toxicities observed were graded according to the Common Toxicity Criteria, version 2.0 (National Cancer Institute, 1998). DLT was defined as any of the following events during the first treatment cycle: grade 3 or 4 anaemia or thrombocytopaenia, febrile neutropaenia grade 3, or grade 4 neutropaenia, grade 3 or 4 biochemical toxicity, grade 2 (or worse) nausea and vomiting despite optimal antiemetic therapy, grade 3 or 4 diarrhoea despite optimal loperamide support, failure to take study medication for 7 days or more within the first treatment cycle due to drug-related toxicity or any CTC grade 3 or 4 nonhaematological toxicity (excluding alopaecia). The MTD was defined as the dose below the dose at which two or three of three, or two or more of six patients experienced DLT during cycle 1.

### Pharmacokinetics

Pharmacokinetic studies were performed in all patients. Ethylenediaminetetraacetic acid blood samples were collected during the first cycle: on day 1 before the first drug intake and 0.5, 1, 2, 4, 6, 8, 12 and 24 h after administration; on days 3, 8 and 15 before the morning drug intake and 2 h after administration; and on day 22 before the morning drug intake and 0.5, 1, 2, 4, 6, 8, 12 and 24 h after administration. Samples were immediately put on ice and centrifuged at 4°C for 10 min at 1500 *g*. The resulting plasma was stored at −70°C until analysis. Plasma samples of the last three patients were not measured as the study was stopped before analysis.

The total plasma concentrations of AZD3409 ester and AZD3409 acid were determined by LC–MS/MS. The plasma samples were treated with dithiothreitol to reduce any disulphide bonds. The sample pretreatment was based on protein precipitation with acidic acetonitrile. Isotopically labelled analogues were used as internal standards. The method has been validated regarding selectivity, accuracy, precision and stability in human plasma. The lower limit of quantification was 3 ng ml^−1^ for both AZD3409 ester and AZD3409 acid, with a 50 *μ*l sample volume.

The following PK parameters were determined, either by direct observation or by noncompartmental analysis (WinNonlin, version 5.0.1; Pharsight, Mountain View, CA, USA): the minimal plasma concentration (*C*_min_), the maximal plasma concentration (*C*_max_), the area under the curve within one dosing interval (AUC_0−*τ*_) and total plasma clearance (Cl/F). Correlations between dose and *C*_max_ levels and between dose and AUC_0−*τ*_ were analysed on day 1 using the nonparametric Spearman's *ρ* (*R*_s_) correlation test. Accumulation was tested with an exact Wilcoxon's rank paired signed test. Linear measures mixed effects models were constructed with SAS (SAS Institute Inc., Cary, NC, USA) to determine the effect of the dose on the trough plasma concentrations of both metabolites. The daily dose, time (days), gender and age were considered to be a fixed effect, and the patient variable was considered to be a random effect. Owing to the distribution of the trough plasma concentration log transformation was applied.

### Pharmacodynamics

Prior to and 2 h after intake on days 1, 2 (only predose), 8 and 22, whole-blood samples of 22.5 ml were collected in heparinised tubes for FTase activity determination in peripheral blood mononuclear cells (PBMCs). Within 30 min, the PBMC fraction was isolated using Accuspin tubes (Sigma). The cell pellet was resuspended in 100 *μ*l lysis buffer (PBS containing 1% (v/v) Triton X-100, 0.5% (w/v) sodium deoxycholate, 0.1% (w/v) sodium dodecylsulphate and 1% (v/v) aprotinin) and stored at −70°C.

FTase activity was measured by adding 5 *μ*l of PBMC lysate, 5 *μ*l of lysis buffer and 10 *μ*l of water to each well. The lysates were preincubated for 10 min prior to the addition of 20 *μ*l of substrate solution (either lamin B or K-Ras). The reaction was initiated by the addition of 10 *μ*l of radiolabelled farnesylpyrophosphate (fpp). The wells were incubated for 40 min at 37°C. The reaction was stopped by the addition of 100 *μ*l of 1.5 M HCl in ethanol. The wells were stored at 4°C for 1 h and then processed using the Tomtec Harvester 96. The filter plates were dried at 37°C for 15 min, 0.35 *μ*l MicroScint was added to each filter well and the radioactivity was counted. Each lysate was analysed in triplicate. Blanks consisted of the same reaction mixture without the substrate.

Linear mixed effects models were constructed to determine the effect of time on the enzyme activity levels. In these models, the daily dose, number of applications, time (days), measurement pre- or postdrug intake, gender and age were considered to be a fixed effect, and the patient variable was considered to be a random effect. Owing to the distribution of the response variables, square root variables were applied. Statistical analysis was performed with SAS. To determine the correlation of the dose level and the enzyme inhibition, a baseline was added to the described models.

Pharmacokinetic-pharmacodynamic correlations between the active acid metabolite and decrease in FTase activity were fitted with WinNonlin software. Only patients with a basal FTase activity that was higher than 10 pg fpp per mg protein per min were included in the model. This cutoff value was arbitrary and was chosen based on the available data (lamin B; mean: 28.0, SD: 14.2, median: 27.8, 25% percentile: 19.1 and K-Ras; mean: 29.3, SD: 17.8, median: 25.6, 25% percentile: 16.7). The decision to set a minimum baseline FTase activity implicates that the model can only be used for patients with a minimum FTase activity of 10 pg fpp per mg protein per min.

## RESULTS

### Patient characteristics

In total, 37 patients have been included in the trial, of whom 32 patients were entered in part A and 5 patients in part B. The number of 37 patients included represents the number of patients screened. Of these 37 patients, 29 patients were found eligible and were dosed in this trial. Patients’ characteristics are presented in Table 1. Median age of the patients was 59 years (range: 38–77 years) and most patients were in good general condition. Patients had various tumour types; colorectal malignancies were most frequent (31% of all dosed patients).

### Treatment

In part A, a total of six dose levels of 500 mg once daily, 1000 mg once daily, 750 mg b.i.d., 1250 mg b.i.d., 1750 mg b.i.d. and 1500 mg b.i.d., respectively, was investigated. In part A, 26 patients fulfilled the eligibility criteria and received medication. In part B, five patients were screened to receive a dose of 250 mg b.i.d. in the fed state. Two patients did not receive medication, because they did not fulfil the eligibility criteria. The median duration of patients on study was 6 weeks (range: 1–15 weeks). In total, 25 patients discontinued study treatment early. The main reasons for discontinuation of the treatment were worsening of the patients’ condition (12 patients) and occurrence of adverse events (10 patients). Two patients were unwilling to continue with the study after 4 and 8 weeks, respectively, and one patient had to stop medication after 3 weeks due to disease progression.

### Toxicity

No significant haematological toxicity was observed during cycle 1 of the study with oral AZD3409. None of the 29 patients who were evaluable experienced leucocytopaenia, thrombocytopaenia or anaemia greater than CTC grade 2 or neutropaenia greater than grade 1. However, in part B, one patient experienced CTC grade 3 thrombocytopaenia after stopping treatment with AZD3409. The patient had advanced breast cancer with distant metastases in the bone, liver and lung. The platelet counts decreased from 109 × 10^9^/l before start of treatment to 24 × 10^9^/l on day 36, 2 weeks after end of the medication.

The drug-related nonhaematological adverse events per patient as a function of the dose are presented in Table 2. Nonhaematological toxicity was reported by many patients and consisted mainly of vomiting (55%), nausea (55%) and diarrhoea (41%). Adverse events were observed both in the once daily and the twice daily regimens. The severity was on average mild to moderate and reversible. However, at the dose level of 1750 mg b.i.d., two patients were unable to take the drug as a consequence of CTC grade 2 vomiting in spite of antiemetics, and, therefore, this toxicity was considered dose limiting. At the next lower dose level of 1500 mg b.i.d., two patients were also unable to continue AZD3409 treatment due to grade 3 diarrhoea and uncontrolled nausea (grade 2), respectively. Both toxicities were dose limiting, and, therefore, the dose was further lowered to 1250 mg b.i.d. In total, three out of six patients at this dose level experienced grade 3 diarrhoea, grade 3 dehydration, grade 2/3 nausea and grade 2 vomiting. These gastrointestinal toxicities were also dose limiting. The MTD of part A was defined as 750 mg b.i.d. in the fasted state.

### Tumour response

Eighteen patients were evaluable for tumour response after 4–14 weeks of treatment. No objective tumour response was observed. Three patients (17%) had stable disease for 47, 48 and 76 days, respectively.

### Pharmacokinetics

**Figure 2:**
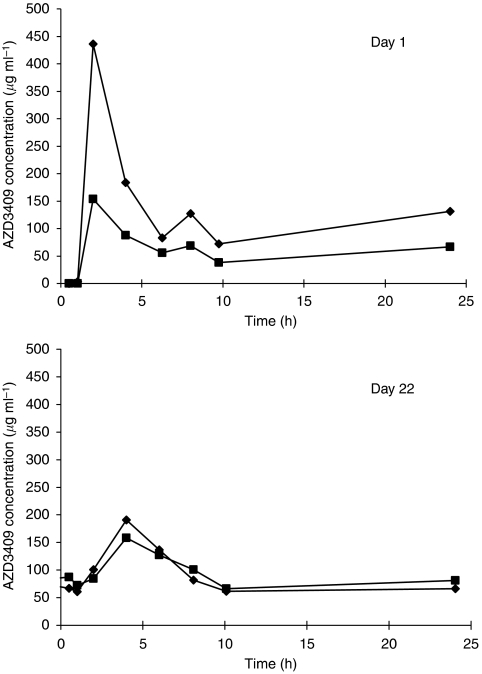
Plasma concentration–time curve of AZD3409 ester (diamond) and AZD3409 acid (square) during day 1 (upper) and day 22 (lower), respectively, in a patient receiving 750 mg b.i.d.

Plasma concentrations of AZD3409 ester and AZD3409 acid were measured in all 26 patients of part A and in none of the patients of part B. The AZD3409 parent drug is rapidly metabolised in the gut and plasma to the AZD3409 ester metabolite and therefore does not appear in the blood. Complete plasma concentration–time curves were obtained for both AZD3409 ester and AZD3409 acid on days 1 and 22 (see Figure 2 for MTD level). The estimated PK parameters for AZD3409 ester and AZD3409 acid at all dose levels are presented in Table 3. Extrapolation of the AUC of AZD3409 ester and AZD3409 acid to infinity exceeded 20% in most cases and therefore AUC values within one dosing interval (AUC_0−*τ*_) were reported. Also, terminal half-lives could not adequately be estimated. Interpatient variability was high and the coefficient of variation ranged between 45 and 103% for the AUC of AZD3409 ester and between 44 and 99% for the AUC of AZD3409 acid on day 1.

A positive correlation between the dose and the AUC and *C*_max_ on day 1 was observed for both AZD3409 ester (*R*_s_=0.52 and 0.43) and AZD3409 acid (*R*_s_=0.52 and 0.43). The plasma concentrations of AZD3409 ester were higher than those of AZD3409 acid. Accumulation was shown for both metabolites, although only significant for AZD3409 acid (*P*=0.006). As a result of the slightly higher acid accumulation, the AUC_ester_/AUC_acid_ ratio changed from 2.5 on day 1 to 2.0 on day 22.

Statistical analysis of the mean predose concentration of AZD3409 ester and AZD3409 acid on days 3, 8, 15 and 22 of the first cycle demonstrated that the trough plasma concentrations increased with the administered dose per day. However, after performing a sensitivity analysis by including the number of applications per day as a fixed effect, no correlation was found between the *C*_min_ and the daily dose. Gender and age did not affect the trough concentrations. A slight increase of *C*_min_ of the acid metabolite over time was observed.

### Pharmacodynamics

**Figure 3:**
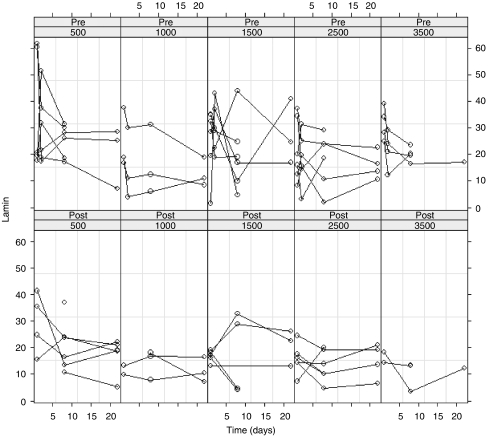
FTase activity (pg fpp per min per mg protein) of each patient over time using lamin B as substrate, separated by pre- (upper) and post (lower) treatment and by daily dose.

FTase activity was measured in 24 patients of part A and 3 patients of part B using both lamin B (Figure 3) and K-Ras as substrates (data not shown). Both patients at the dose level of 1500 mg b.i.d. were excluded for statistical analysis due to lack of data. For the statistical analysis, no differentiation was made between parts A and B. Enzyme inhibition was observed in all patients, although on different days, except in patient 3 (dose level 500 mg day^-1^) and patient 24 (dose level 750 mg b.i.d.). Statistical analysis showed that, as expected, on each day the degree of FTase inhibition after intake was significantly higher than prior to intake of the drug (Table 4). Over time, there was no significant drop of K-Ras values for FTase activity, whereas the lamin B substrate values significantly decreased (*P*=0.02). In addition, it was investigated whether an increase in daily dose affected the FTase inhibition. It was shown that the FTase activity with the K-Ras substrate significantly decreased (*P*=0.03), but no effect was observed on the FTase activity with the lamin B substrate. With regards to the other variables, patient age, gender and number of doses per day did not influence the outcome. When a correction for the baseline values of FTase activity is included in the model, the K-Ras values for FTase activity were not significantly decreased over time nor with increasing daily dose. In contrast, the lamin B values for FTase activity were decreased both over time (*P*=0.04) and with increasing daily dose (*P*=0.02).

**Figure 4:**
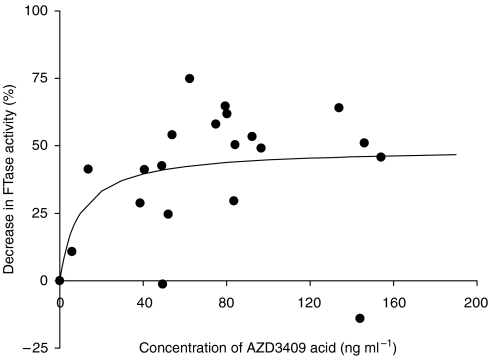
*E*_max_ model of the AZD3409 acid plasma concentrations and the matching relative decrease in lamin B FTase activity in PBMC lysate on day 1. Blood samples were taken 2 h after intake of AZD3409. The maximal level of FTase inhibition was estimated at 49%. The concentration, at which 50% of the maximal effect is reached, was estimated at 9.5 ng ml^−1^. Correlation *E*_max_/EC_50_=0.85, bias=−0.18% and precision=21.1%.

To reveal PK/PD relationships, the AZD3409 acid plasma concentrations of 22 patients of part A were plotted against the proportional decrease in FTase activity at equal sampling times. Three and two patients for K-ras and lamin B, respectively, who had an initial FTase activity lower than 10 pg fpp per mg protein per min were excluded. At first sight, there was no relationship. However, when only the data of day 1 were used for this analysis, we found that the lamin B data could be fitted to an *E*_max_ model (Figure 4) using the following equation: *E*=(*E*_max_^*^C)/(*C*+EC_50_). The maximal effect (*E*_max_) was estimated at 49% (±11) and the EC_50_ was 9.5 (±13.9)  ng ml^−1^. The K-Ras data could not be fitted due to high scatter.

**Figure 5:**
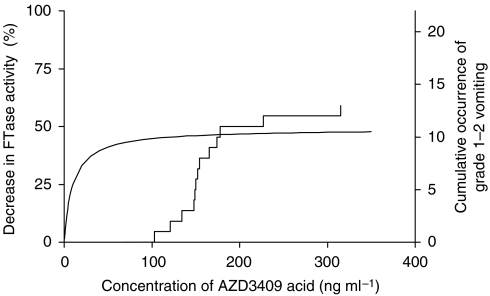
The cumulative occurrence of CTC grade 1–2 vomiting *vs* the maximal AZD3409 acid plasma concentration during days 1–22. The *E*_max_ curve from Figure 4 is also shown. The maximal biological effect is reached at lower plasma concentrations than the toxicity of vomiting.

Figure 5 presents the *E*_max_ model for lamin B as well as the *C*_max_ in cycle 1 *vs* the cumulative occurrence of grade 1–2 vomiting in cycle 1 (*n*=22). The time at which FTase activity was determined was approximately 2 h after administration, and the mean time at which *C*_max_ was reached was 2.7 h. At approximately 100 ng ml^−1^, 90% of the maximal biological effect is reached, whereas the chance to suffer from vomiting due to the medication is below 15% at maximal plasma concentrations up to 140 ng ml^−1^. Similar results were obtained for nausea toxicity (data not shown).

## DISCUSSION

The presented phase I trial demonstrates that continuous oral dosing of the novel DPTI AZD3409 is feasible. The maximum administered dose was 1750 mg b.i.d. The MTD was defined in part A as 750 mg b.i.d. in the fasted state.

AZD3409 is a double prodrug, which complicates its pharmacokinetic analysis. The drug is rapidly metabolised to AZD3409 ester when it enters the blood stream. Hydrolysis of the ester group of AZD3409 ester to form AZD3409 acid is a major pathway in the liver (unpublished data). As expected, both metabolites but not the parent drug could be detected in plasma. The PK studies in our trial showed a positive correlation between the AZD3409 dose and the parameters AUC_0−*τ*_ and *C*_max_ of both metabolites on day 1. However, interpatient variability was considerable at all dose levels. On day 22, no significant correlation could be established between the dose of AZD3409 and the parameters AUC_0−*τ*_ and *C*_max_ of both metabolites. This could be due to the lower number of patient data that were available on day 22 to estimate the PK parameters. Otherwise, it is possible that a large interpatient variability is observed as a result of the biological processes in the blood stream and body. Differences in plasma levels may also be caused by differences in plasma protein binding as described below.

In this trial, mean trough ‘total’ AZD3409 concentrations in the twice daily dosing levels ranged between 31.3 and 434 ng ml^−1^ (assuming that AZD3409 ester and AZD3409 acid are the main metabolites in plasma). *In vitro*, AZD3409 inhibits cell proliferation of tumour cells at IC_50_ values between 0.05 and 5 *μ*M, which corresponds to 35–3500 ng ml^−1^ (Kelly *et al*, 2005). Hence, the achieved plasma concentrations are likely within the active range. Nevertheless, a considerable fraction might be bound to plasma proteins. Estimation of the plasma protein binding of AZD3409 is very complex, because both AZD3409 ester and AZD3409 acid have a free thiol group that can form covalent bonds with proteins as disulphides. This is atypical of protein binding, which is normally a physical, rather than chemical association. It has been suggested that glutathione reduces the measured protein binding, possibly acting as an alternative partner in the formation of a disulphide. In addition to forming disulphides with endogenous thiol-containing compounds, such as proteins and glutathione, the free thiol-containing metabolites may form disulphides with each other. Extraction of AZD3409 ester and AZD3409 acid from plasma is performed after reduction with dithiothreitol, which means that the concentrations determined may have existed in plasma as a complex mixture of disulphides. Therefore, at this stage, it has not been established which fraction of analytes present in plasma represents active drug (unpublished data).

Estimated clearance/F of both metabolites was very high, which could be explained by low absorption of the parent drug from the gastrointestinal tract. In a pilot mass balance study in rats, only 1% of the given radioactivity was excreted in the urine supporting low absorption of orally dosed AZD3409 from the gut (Kelly *et al*, 2005).

The AUC_ester_/AUC_acid_ ratio was slightly decreased on day 22. This is probably due to the higher accumulation of AZD3409 acid. Another explanation might be induction of esterases that convert AZD3409 ester into the acid form. However, *in vivo*, no biologically significant changes were observed in cytochrome *P*450 content or enzyme activity after once daily, oral dosing to rats for 28 days (unpublished data).

*In vitro*, AZD3409 is a potent inhibitor of FTase. In this trial, PD analysis showed that FTase activity in PBMCs could significantly be inhibited by AZD3409 during 21 days of oral administration. At predose time points after repeated dosing, the AZD3409 acid concentrations were near the limit of quantitation, while the FTase inhibition in PBMCs persisted. This indicates that continuous inhibition of FTase can be accomplished by daily dosing of AZD3409. Furthermore, the plasma concentrations of AZD3409 acid on day 1 could be adequately correlated to the relative decrease of FTase activity by an *E*_max_ model. Two patients were excluded from this model, because they had a low FTase activity at the start of the study (<10 pg fpp per mg protein per min) compared with all other patients (mean=31 pg fpp per mg protein per min). A very low basal activity implies that inhibition cannot be measured. The maximal decrease in activity was estimated at 49%, indicating that FTase could not completely be inhibited. Similar inhibition values were found in studies with single FTase inhibitors (Cohen *et al*, 2003; Alsina *et al*, 2004; Cortes *et al*, 2005; Tabernero *et al*, 2005). In the Calu-6 xenograft model, inhibition of FTase by 66% was associated with a reduction of the tumour volume of 15% (unpublished data). It has to be established whether the level of inhibition of FTase that was seen in PBMCs in this trial will translate into meaningful antitumour activity. Only 3 out of 16 patients had stable disease after approximately 2 months. The EC_50_ value in the *E*_max_ model of the lamin B substrate was estimated at 15.2 ng ml^−1^ AZD3409 acid in plasma. This suggests that the mean trough total AZD3409 acid concentrations in the twice daily dosing levels (36.3–81.9 ng ml^−1^, see also Figure 2) were high enough to cause at least 75% of the maximally achievable FTase inhibition. It is unknown whether there is a relationship between the observed inhibition of FTase activity as documented in PBMCs and FTase activity in tumour cells.

The occurrence of grade 1–2 vomiting was plotted against the *C*_max_, because it was assumed that this drug-related toxicity is mainly determined by the maximal plasma concentration. The results indicate that maximal FTase inhibition is reached at exposure levels that are lower than levels associated with induction of significant vomiting.

Although the PK/PD analysis of AZD3409 acid is preliminary, the approach may be meaningful for selecting the optimal dose of the drug. Currently, it is unclear whether compounds that are not directly cytotoxic, but target a biological pathway should be dosed based on their maximal toxicity or on their maximal biological effect (Workman, 2002; Garrett *et al*, 2003; Appels *et al*, 2005). We showed that 90% of the maximal biological effect was reached at AZD3409 acid plasma concentrations that were not toxic.

In conclusion, we have demonstrated that chronic oral dosing with AZD3409 is feasible. We proved the concept that AZD3409 can inhibit FTase activity in surrogate cells, that is, PBMCs. It is, however, unclear to what level FTase activity is inhibited in tumour tissue and whether the observed inhibition of FTase in PBMCs will be sufficient for antitumour activity. In addition, PD studies revealed that the maximal FTase inhibition is reached at AZD3409 acid plasma concentrations at which the occurrence of drug-related toxicity is low. The estimated maximal FTase activity is approximately 50% and future studies are warranted to determine the antitumour activity of AZD3409 when dosed twice daily. This study supports the rationale to implement biological effect studies in clinical trials with biologically active anticancer drugs to define the optimal dosing regimen.

## Figures and Tables

**Figure 1:**
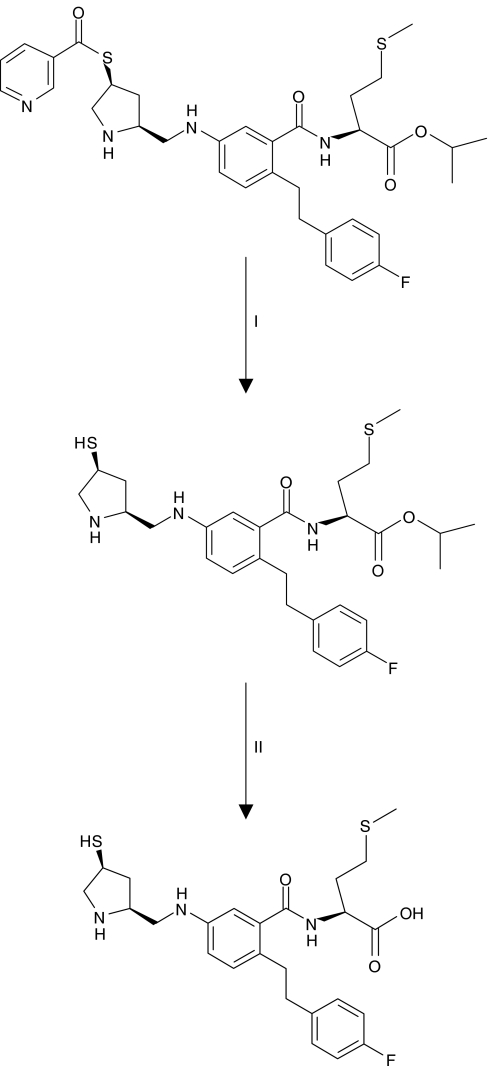
Chemical structures of AZD3409. Reaction I: AZD3409 is broken down by esterases in the plasma to from its major circulating metabolite AZD3409 ester. Reaction II: After penetration into the cell, AZD3409 ester is converted into the active drug AZD3409 acid under the action of intracellular esterases.

**Table 1:** Patient characteristics

	**No. of patients**
Total	29
*Sex*	
Male	13
Female	16
	
*Age (years)*	
Median	58
Range	38–77
	
*Performance status*	
0	13
1	15
2	1
Unknown	0
	
*Primary tumour location*	
Colorectal	9
Breast	6
Oesophagus	3
Skin/soft tissue	3
Pancreas	1
Kidney	1
Liver	1
Other	3
Unknown	2
	
*Previous therapy*	
Chemotherapy	29
Radiotherapy	15
Other therapy	10

**Table 2:** Possibly or definitely drug-related nonhaematological toxicity in cycle 1 that was observed in more than 10% of all treated patients

**Adverse event**	**Grade**	**Dose (mg)**							**Total**
**Number of patients**		**500**	**1000**	**750 b.i.d.**	**1250 b.i.d.**	**1500 b.i.d.**	**1750 b.i.d.**	**250 b.i.d.**	***n* (%)**
		**3**	**3**	**8**	**6**	**2**	**4**	**3**	**29**
Abdominal pain	1–2			1	2				3 (10)
Anorexia	1–2	1		2	1		2		6 (21)
Diarrhoea	1–2		3	3	2		4		12 (41)
	3–4				1	1			2 (7)
Fatigue	1–2	1		2	1		1		5 (17)
Nausea	1–2	1	2	5	3	1	3	1	16 (55)
	3–4				1				1 (3)
Vomiting	1–2	1	2	4	3	1	3	2	16 (55)

**Table 3:** Pharmacokinetic parameters of AZD3409 ester and AZD3409 acid on days 1 and 22 after administration of AZD3409

	**Day 1**			**Day 22**				
**Dose**	***N***	***C*_max_ (ng ml^−1^)**	**AUC_0−_**_***τ***_ **(ng h ml^−1^)**	***N***	***C*_max_ (ng ml^−1^)**	**Cl/F (l h^−1^)**	**AUC_0−_**_***τ***_ **(ng h ml^−1^)**	**Ratio AUC_0−_**_***τ***_ **day 22/day 1**
*AZD3409 ester*								
500	3	82.1 (70.0)	395 (407)	2	[229–257]	[263–308]	[1230–1465]	[1.42–8.31]
1000	3	318 (254)	1545 (788)	3	296 (96.9)	319 (172)	1934 (279)	1.54 (0.86)
750 b.i.d.	8	245 (152)	1066 (816)	3	298 (127)	309 (151)	1724 (656)	7.03 (8.46)
1250 b.i.d.	6	338 (190)	1777 (972)	4	366 (132)	474 (231)	2123 (788)	1.28 (0.70)
1500 b.i.d.	2	[227–315]	[1456–1627]	—	—	—	—	—
1750 b.i.d.	4	362 (154)	1693 (757)	1	445	1145	1098	0.97
								
*AZD3409 acid*								
500	3	33.6 (25.4)	160 (159)	2	[95.9–111]	[465–488]	[712–775]	[2.07–10.1]
1000	3	78.6 (24.6)	512 (229)	3	106 (15.5)	710 (148)	904 (113)	2.10 (1.19)
750 b.i.d.	8	93.8 (55.7)	468 (341)	3	138 (30.7)	473 (155)	934 (220)	9.29 (11.2)
1250 b.i.d.	6	119 (64.9)	677 (322)	4	168 (77.3)	785 (311)	1230 (704)	1.95 (1.05)
1500 b.i.d.	2	[83.5–148]	[458–749]	—	—	—	—	—
1750 b.i.d.	4	140 (62.6)	751 (260)	1	165	1706	466	1.23

—=no data available; AUC_0−***τ***_=AUC within one dosing interval.

Represented as mean and (standard deviation) or as [absolute values] when *n*<3.

**Table 4:** Statistical significance of dosing variables on the inhibition of FTase activity in PBMCs of patients treated with different doses of AZD3409

	**Estimate**		***P*-value**	
**Variable**	**Baseline not included**	**Baseline included**	**Baseline not included**	**Baseline included**
*Lamin B*				
Pre/post treatment	0.62	0.63	<0.001	<0.001
Time	−0.025	−0.023	0.02	0.04
Total dose	−0.0002	−0.0004	0.32	0.02
Doses per day	0.18	0.67	0.71	0.05
Age	0.00003	−0.0053	1.0	0.67
Sex	0.61	0.17	0.09	0.49
Baseline	NA	0.38	NA	<0.001
				
*K-Ras*				
Pre/post treatment	0.71	0.68	<0.001	<0.001
Time	−0.016	−0.013	0.10	0.18
Total dose	−0.00073	−0.00034	0.03	0.09
Doses per day	−0.062	0.30	0.94	0.49
Age	0.035	−0.0066	0.28	0.72
Sex	0.24	−0.048	0.68	0.87
Baseline	NA	0.56	NA	<0.001

NA=not applicable.

## References

[bib1] Alsina M, Fonseca R, Wilson EF, Belle AN, Gerbino E, Price-Troska T, Overton RM, Ahmann G, Bruzek LM, Adjei AA, Kaufmann SH, Wright JJ, Sullivan D, Djulbegovic B, Cantor AB, Greipp PR, Dalton WS, Sebti SM (2004) Farnesyltransferase inhibitor tipifarnib is well tolerated, induces stabilization of disease, and inhibits farnesylation and oncogenic/tumor survival pathways in patients with advanced multiple myeloma. Blood 103(9): 3271–3277 14726402 10.1182/blood-2003-08-2764

[bib2] Appels NMGM, Beijnen JH, Schellens JHM (2005) Development of farnesyl transferase inhibitors; a review. Oncologist 10(8): 565–578 16177281 10.1634/theoncologist.10-8-565

[bib3] Bos JL (1989) ras oncogenes in human cancer: a review. Cancer Res 49(17): 4682–4689 2547513

[bib4] Brunner TB, Hahn SM, Gupta AK, Muschel RJ, McKenna WG, Bernhard EJ (2003) Farnesyltransferase inhibitors: an overview of the results of preclinical and clinical investigations. Cancer Res 63(18): 5656–5668 14522880

[bib5] Casey PJ, Seabra MC (1996) Protein prenyltransferases. J Biol Chem 271(10): 5289–5292 8621375 10.1074/jbc.271.10.5289

[bib6] Cohen SJ, Ho L, Ranganathan S, Abbruzzese JL, Alpaugh RK, Beard M, Lewis NL, McLaughlin S, Rogatko A, Perez-Ruixo JJ, Thistle AM, Verhaeghe T, Wang H, Weiner LM, Wright JJ, Hudes GR, Meropol NJ (2003) Phase II and pharmacodynamic study of the farnesyltransferase inhibitor R115777 as initial therapy in patients with metastatic pancreatic adenocarcinoma. J Clin Oncol 21(7): 1301–1306 12663718 10.1200/JCO.2003.08.040

[bib7] Cortes J, Faderl S, Estey E, Kurzrock R, Thomas D, Beran M, Garcia-Manero G, Ferrajoli A, Giles F, Koller C, O’Brien S, Wright J, Bai SA, Kantarjian H (2005) Phase I study of BMS-214662, a farnesyl transferase inhibitor in patients with acute leukemias and high-risk myelodysplastic syndromes. J Clin Oncol 23(12): 2805–2812 15728224 10.1200/JCO.2005.09.005

[bib8] Crul M, de Klerk GJ, Beijnen JH, Schellens JHM (2001) Ras biochemistry and farnesyl transferase inhibitors: a literature survey. Anticancer Drugs 12(3): 163–184 11290863 10.1097/00001813-200103000-00001

[bib9] Delarue FL, Adnane J, Joshi B, Blaskovich MA, Wang DA, Hawker J, Bizouarn F, Ohkanda J, Zhu K, Hamilton AD, Chellappan S, Sebti SM (2007) Farnesyltransferase and geranylgeranyltransferase I inhibitors upregulate RhoB expression by HDAC1 dissociation, HAT association and histone acetylation of the RhoB promoter. Oncogene 26(5): 633–640 16909123 10.1038/sj.onc.1209819

[bib10] Garrett MD, Walton MI, McDonald E, Judson I, Workman P (2003) The contemporary drug development process: advances and challenges in preclinical and clinical development. Prog Cell Cycle Res 5: 145–158 14593708

[bib11] Kelly J, Dominguez-Escrig J, Leung HY, Stephens TC, Neal DE, Davies BR (2005) The prenyltransferase inhibitor AZD3409 has anti-tumor activity in preclinical models of urothelial carcinoma. Proc Am Assoc Cancer Res 46; abstract 5962

[bib12] Leary AF, Sirohi B, Johnston SR (2007) Clinical trials update: endocrine and biological therapy combinations in the treatment of breast cancer. Breast Cancer Res 9(5): 112 17980056 10.1186/bcr1763 PMC2242654

[bib13] National Cancer Institute (1998) Guidelines for reporting adverse drug interactions. Division of Cancer Treatment. National Cancer Institute: Bethesda, MD

[bib14] Sebti SM, Adjei AA (2004) Farnesyltransferase inhibitors. Semin Oncol 31(1 Suppl 1): 28–39 10.1053/j.seminoncol.2003.12.012 14981578

[bib15] Stephens TC, Wardleworth MJ, Matusiak ZS, Ashton SE, Hancox UJ, Bate M, Ferguson R, Boyle T (2003) AZD3409, a novel, oral, protein prenylation inhibitor with promising preclinical antitumor activity. Proc Am Assoc Cancer Res 44: R4870

[bib16] Tabernero J, Rojo F, Marimón I, Voi M, Albanell J, Guix M, Vázquez F, Carulla J, Cooper M, Andreu J, Van Vreckem A, Bellmunt J, Manne V, Manning JA, Garrido C, Felip E, Del Campo JM, García M, Valverde S, Baselga J (2005) Phase I pharmacokinetic and pharmacodynamic study of weekly 1-h and 24-h infusion BMS-214662, a farnesyltransferase inhibitor, in patients with advanced solid tumors. J Clin Oncol 23(11): 2521–2533 15710949 10.1200/JCO.2005.00.398

[bib17] Tamanoi F, Kato-Stankiewicz J, Jiang C, Machado I, Thapar N (2001) Farnesylated proteins and cell cycle progression. J Cell Biochem Suppl 37: 64–70 11842430 10.1002/jcb.10067

[bib18] Therasse P, Arbuck SG, Eisenhauer EA, Wanders J, Kaplan RS, Rubinstein L, Verweij J, Van Glabbeke M, van Oosterom AT, Christian MC, Gwyther SG (2000) New guidelines to evaluate the response to treatment in solid tumors. European Organization for Research and Treatment of Cancer, National Cancer Institute of the United States, National Cancer Institute of Canada. J Natl Cancer Inst 92(3): 205–216 10655437 10.1093/jnci/92.3.205

[bib19] Whyte DB, Kirschmeier P, Hockenberry TN, Nunez-Oliva I, James L, Catino JJ, Bishop WR, Pai JK (1997) K- and N-Ras are geranylgeranylated in cells treated with farnesyl protein transferase inhibitors. J Biol Chem 272(22): 14459–14464 9162087 10.1074/jbc.272.22.14459

[bib20] Workman P (2002) Challenges of PK/PD measurements in modern drug development. Eur J Cancer 38(16): 2189–2193 12387843 10.1016/s0959-8049(02)00395-7

